# Supplementary material for: Identification of Novel Fusion Transcripts in High Grade Serous Ovarian Cancer
Source: Int J Mol Sci. 2021 Apr 30;22(9):4791. doi: 10.3390/ijms22094791 (PMC8125626; doi:10.3390/ijms22094791)
Supplement: Supplementary file 1 [file ijms-22-04791-s001.zip › ijms-1180631-Supplemental/Supplemental/Supplementary Table S2 - Chemo-response patient characteristics.pdf]

Supplementary Table S1: Patient clinical and pathological characteristics based on their response to therapy.
Univariate analysis with logistic regression was used to assess differences between patients that responded or not responded to initial chemotherapy.

			Responders	Non-responders	p-value
			N=50	N=38	
**Age**			56	64	0.009*
**Charlson Comorbidity Index**	1-3		8	4	0.045*
	4-6		29	21	
	> 6		1	6	
**Stage**	3		37	25	0.080
	4		6	11	
**Disease in Upper abdomen (Other than Omentum) by Imaging**	Yes	Large Bowel (N=4)	28	29	0.051
		Porta - Hepatis (N=4)			
		Mesenteric Mets (N=3)			
		Other (N=22)			
	No		22	9	
**Disease in the Chest by Imaging**	Yes	Chest (N=3)	5	0	0.992
		Pleural effusion (N=4)			
	No		38	36	
**Grade**	2		8	11	0.146
	3		35	22	
**Residual disease after surgery**	Microscopic		8	3	0.200
	Macroscopic		35	33	
	Optimal (<1 cm)		37	20	0.039*
	Suboptimal (>1 cm)		13	18	
**Removal of Pelvic LN**	Yes		6	4	0.706
	No		37	32	
**Removal of Para-Aortic LN**	Yes		3	3	0.821
	No		40	33	
**Surgical complexity score****	Low		24	26	0.062
	Intermediate		19	8	
	High		0	2	
**Neoadjuvant Chemotherapy**	Yes		2	10	0.008*
	No		48	28	
**Number of Cycles delivered**	< 6		1	7	0.194
	≥ 6		42	29	
**Dose Dense Chemotherapy**	Yes		1	1	0.899
	No		42	35	

*Statistically significant (p-value<0.05).
**Modification of Mayo complexity index: we did not have an entry of peritoneal or abdominal stripping; and rectosigmoidectomies with anastomosis were considered in our data collection as large bowel resections with anastomosis.
