# Supplementary material for: Identification of Novel Fusion Transcripts in High Grade Serous Ovarian Cancer
Source: Int J Mol Sci. 2021 Apr 30;22(9):4791. doi: 10.3390/ijms22094791 (PMC8125626; doi:10.3390/ijms22094791)
Supplement: Supplementary file 1 [file ijms-22-04791-s001.zip › ijms-1180631-Supplemental/Supplemental/Supplementary Table S3 - Significant fusion genes survival.pdf]

Supplementary Table S3: Fusion genes significant associated with survival in the univariate analysis
Univariate analysis with Cox proportional Hazard ratio was used to assess differences in survival for all fusion genes. Only showing those significant with a p-value < 0.05.

**Fusion genes**	**OR**	**95% CI**	**p-value**
**Z68871.1--LINC00630**	1.96	1.30, 2.95	0.001
**ZBTB8OS--AC090627.1**	95.49	5.97, 1526.75	0.001
**ARL17A--KANSL1**	31.32	3.26, 301.16	0.003
**TOGARAM1--FANCM**	31.32	3.26, 301.16	0.003
**UBE2F--LRRFIP1**	31.32	3.26, 301.16	0.003
**AC007952.4--RN7SL2**	1.23	1.07, 1.43	0.005
**AP3D1--ARHGDIA**	23.34	2.61, 208.85	0.005
**ARHGAP1--CKAP5**	23.34	2.61, 208.85	0.005
**DOT1L--GCGR**	23.34	2.61, 208.85	0.005
**FAM20C--AC093627.4**	4.83	1.62, 14.45	0.005
**INPP5B--PLEKHO1**	23.34	2.61, 208.85	0.005
**MTCH2--AGBL2**	23.34	2.61, 208.85	0.005
**FAM98B--FRMD5**	15.35	1.85, 127.48	0.011
**LUC7L--AXIN1**	15.35	1.85, 127.48	0.011
**MAGED2--ZFAT**	3.92	1.36, 11.29	0.011
**NFE2L1--PNPO**	15.35	1.85, 127.48	0.011
**TMCC1--CD96**	15.35	1.85, 127.48	0.011
**NRIP1--AJ009632.2**	1.90	1.13, 3.21	0.016
**PACS1--HAUS3**	13.04	1.60, 106.00	0.016
**PGM2L1--POLD3**	13.04	1.60, 106.00	0.016
**SMARCA4--ZNF700**	13.04	1.60, 106.00	0.016
**AUTS2--INO80C**	11.32	1.42, 90.50	0.022
**JMJD1C--CCNYL1**	11.32	1.42, 90.50	0.022
**PARP4--BAGE2**	11.32	1.42, 90.50	0.022
**PRSS42P--PRSS50**	11.32	1.42, 90.50	0.022
**PSPC1--ZMYM5**	11.32	1.42, 90.50	0.022
**SRGAP3--AC068631.1**	11.32	1.42, 90.50	0.022
**ZNF609--SNX1**	11.32	1.42, 90.50	0.022
**AC004475.1--PRPF6**	9.98	1.26, 78.80	0.029
**BTBD10--TEAD1**	2.15	1.08, 4.29	0.029
**NFKBIB--TEAD1**	9.98	1.26, 78.80	0.029
**UBA2--RAD51B**	3.16	1.12, 8.88	0.029
**CC2D1A--CPNE8**	8.91	1.14, 69.59	0.037
**CHTOP--PCAT1**	8.91	1.14, 69.59	0.037
**FBXO34--SORCS3**	8.91	1.14, 69.59	0.037
**GRIN2A--C16orf72**	8.91	1.14, 69.59	0.037
**PCAT1--C1orf210**	8.91	1.14, 69.59	0.037

**PIK3R3--ANKFN1**	8.91	1.14, 69.59	0.037
**RB1CC1--LINC02091**	8.91	1.14, 69.59	0.037
**TRMT1--CPA4**	8.91	1.14, 69.59	0.037
**AF235103.3--ZNF250**	8.03	1.04, 62.22	0.046
**LINC02408--CAND1**	8.03	1.04, 62.22	0.046
**MECOM--AC116337.3**	8.03	1.04, 62.22	0.046
**TRAPPC3--MAP7D1**	8.03	1.04, 62.22	0.046
