# Supplementary material for: Identification of Novel Fusion Transcripts in High Grade Serous Ovarian Cancer
Source: Int J Mol Sci. 2021 Apr 30;22(9):4791. doi: 10.3390/ijms22094791 (PMC8125626; doi:10.3390/ijms22094791)
Supplement: Supplementary file 1 [file ijms-22-04791-s001.zip › ijms-1180631-Supplemental/Supplemental/Supplementary Table S4 - Validation of fusion genes survival.pdf]

Supplementary Table S4: Survival analysis of the transcripts significant in the multivariate analysis observed with FusionCatcher. Multivariate analysis with Cox proportional Hazard ratio (HR) was used to assess differences in survival for all fusion genes; CI: confidence interval.*Statistically significant.

	**N**	**Median**	**HR**	**p-value**	**95% CI**
ARL17A--KANSL1	15	20.6	1.79	0.054	0.99, 3.26
CC2D1A--CPNE8	1	7.4	6.93	0.074	0.83, 57.95
FAM98B--FRMD5	1	6.1	22.32	0.005*	2.58, 192.82
NRIP1--AJ009632.2	1	6.3	19.01	0.007*	2.25, 160.87
