# Supplementary material for: Identification of Novel Fusion Transcripts in High Grade Serous Ovarian Cancer
Source: Int J Mol Sci. 2021 Apr 30;22(9):4791. doi: 10.3390/ijms22094791 (PMC8125626; doi:10.3390/ijms22094791)
Supplement: Supplementary file 1 [file ijms-22-04791-s001.zip › ijms-1180631-Supplemental/Supplemental/Supplementary Table S5 - Fusion Transcript Size Analysis.pdf]

Supplementary Table S5: Fusion Transcript Size Analysis. T test of fusion transcript sizes, cancer versus normal fallopian tubes. Bp = base pairs.

	Cancer Fusion Sizes	*Fallopian Tube Transcript Sizes*
Mean	6809619.163 bp	54740.57426 bp
Variance	3.69277E+15	8289709984
Observations	1546	102
Hypothesized Mean Difference	0	
df	1545	
t Stat	4.370574985	
P(T<=t) one-tail	6.6094E-06	
t Critical one-tail	1.645840481	
P(T<=t) two-tail	1.32188E-05	
t Critical two-tail	1.961500618	
